# JMM Profile: Mycoplasma genitalium: a small, yet significant pathogen

**DOI:** 10.1099/jmm.0.001984

**Published:** 2025-04-04

**Authors:** Tia Rudman, Richard S. Rowlands, Jorgen S. Jensen, Michael L. Beeton, On behalf of the ESCMID Study Group for Mycoplasma and Chlamydia Infections (ESGMAC)

**Affiliations:** 1 Microbiology and Infection Research Group, Department of Biomedical Sciences, Cardiff Metropolitan University, Cardiff, UK; 2 School of Biosciences, Cardiff University, Cardiff, UK; 3 Statens Serum Institut, Copenhagen, Denmark

**Keywords:** fluoroquinolones, macrolide resistance, non-gonococcal urethritis

## Abstract

*Mycoplasma genitalium* is characterized by a small genome and a lack of a cell wall, contributing to its unique biology. It is associated with reproductive tract infections, including non-gonococcal urethritis and pelvic inflammatory disease. It is nearly as common as chlamydia in most studies from high-income countries. The emergence of antimicrobial resistance in *M. genitalium* raises concern about the long-term efficacy of current therapeutic strategies. Understanding its genomic intricacies and pathogenic mechanisms is crucial for developing targeted interventions to address the growing public health impact of this elusive microbe.

## Graphical abstract

*Mycoplasma genitalium* infections are enabled by multiple virulence factors, both intracellular and expressed on the cholesterol-containing phospholipid membrane. Intracellular accessory proteins form the tip structure, which adheres to epithelial cells *in vivo* via the transmembrane adhesion complex of Nap proteins, P110 and P140. Protein M binds to the Fab region of immunoglobulins with a high affinity to both λ and κ chains, enabling immune system evasion. Membrane lipoproteins and extracellular polymeric substances help in biofilm formation, which is thought to facilitate infection persistence.

## Historical perspective

In the 1970s, clinicians treating patients with non-gonococcal urethritis (NGU) noticed positive responses to tetracycline treatment in some men despite the absence of detectable bacteria in their urethras [1]. In 1980, urethral swabs from patients presenting with NGU yielded two distinct mycoplasmas designated G37 and M30 after prolonged incubation [2]. These isolates, later identified as *Mycoplasma genitalium*, were serologically different from other *Mycoplasmataceae* but most closely related to *M. pneumoniae* [2]. Due to the fastidious nature of the species, further studies required the use of PCR [3]. In the 1990s and early 2000s, the clinical significance of *M. genitalium* became clear, linking it to urethritis in men and women [4,6] and cervicitis [7,8], and molecular typing showed it to be a sexually transmitted infection (STI) [9]. In the 2000s, growing concern about antibiotic resistance in *M. genitalium* led to increased research into its resistance patterns and the development of effective treatment strategies [10,12].

## Clinical presentation(s)

Those infected with *M. genitalium* are often asymptomatic (40–75%) [13]. Female patients, however, may present with vaginal discharge, dysuria, urinary urgency, intermenstrual bleeding and lower abdominal pain as a sign of pelvic inflammatory disease (PID) [14]. In men, symptomatic *M. genitalium* most often presents as urethritis, but epididymitis, balanoposthitis and chronic prostatitis, as well as rectal infections in men who have sex with men (MSM), have been described [14]. Persistent and chronic infections have been documented [15,16].

## Microbial characteristics

### Phenotypic features

*M. genitalium*, like other Mollicutes, lacks a cell wall, is exceptionally small at 200–300 nm, making it one of the smallest known self-replicating microorganisms [17], ferments glucose, requires specific sterols for growth [2] and displays gliding motility, although the exact mechanism is not fully understood [18].

### Genotypic features

*M. genitalium* possesses a small genome of ~0.58 Mb with a 32% G+C content and limited biosynthetic capabilities [19,20]. Its reduced metabolic pathways necessitate host resources for essential metabolites [21]. Although *M. genitalium* is pathogenic, it has fewer virulence factors compared with other urogenital tract pathogens [22]. To date, no plasmids have been identified in *M. genitalium*. The transferable tetracycline resistance gene, *tetM*, which has been documented in other genital mycoplasmas [23,24], has not been detected in *M. genitalium* despite the poor clinical efficacy of doxycycline [11].

## Laboratory confirmation and safety

### Specimen type

Although *M. genitalium* has been detected in a variety of urogenital and extragenital specimen types, the recommendation [25] is to use sample collection methods already in place for *C. trachomatis* and *N. gonorrhoeae* sampling, i.e. first void urine from men, which may be self-collected [26,27]. In women, a vaginal swab (physician- or self-collected) provides the best performance if only one sample is taken [27]. Rectal samples can be considered in MSM where as many as 70% of the infections will be missed if this site is not sampled [28]. However, testing from this site is only indicated in men with symptomatic proctitis where other aetiologies have been excluded due to the high risk of combined macrolide and quinolone resistance in MSM. Rectal infection in women at risk is not uncommon [29].

### Laboratory confirmation

Due to its slow growth rate and fastidious growth requirements for a sterol and nucleotide-containing media, primary axenic culture is seldom undertaken. Incubation with Vero cells increases the chances of primary isolation, and antimicrobial susceptibility testing can be performed on the cell-culture-grown organisms [12]. Culture and isolation of *M. genitalium* have an important role in comparing emerging novel phenotypic resistance and the association with genotypic resistance mechanisms, which may be missed with contemporary molecular assays. Nucleic acid amplification tests (NAATs) are the only available diagnostic method. These can be laboratory-developed PCRs or commercially available assays and have been extensively described in the literature [30]. Detection of macrolide resistance mutations (MRMs) in domain V of the 23S rRNA gene is essential for proper patient management but is unfortunately not an integrated part of the high-throughput commercially available NAATs.

### Laboratory safety

All human *Mycoplasma* sp. are regarded as Hazard Group 2 by the UK Health and Safety Executive. Handling of samples should be undertaken within containment Biosafety Level 2/Containment Level 2, unless suspicion of a pathogen of greater hazard grouping status [31].

## Treatment and resistance

### Treatment

Treatment options are limited due to the lack of a cell wall. Mycoplasmas are typically susceptible to macrolide, fluoroquinolone and tetracycline antibiotics. The European and British Association for Sexual Health and HIV (BASHH) guidelines are available for the management of *M. genitalium* infection [25,32]. In all guidelines, diagnostic testing is only recommended for patients with symptoms, due to growing issues with resistance. In the situation of uncomplicated *M. genitalium* infection without MRMs or resistance testing, BASHH suggests doxycycline 100 mg twice a day for 7 days followed by azithromycin 1 g orally as a single dose then 500 mg orally once daily for 2 days. If treatment with azithromycin fails or if the organism is known to be macrolide-resistant, treat with 400 mg of oral moxifloxacin for 7 days [32]. In the European guidelines, treatment of uncomplicated, macrolide-susceptible infection is azithromycin 500 mg day 1 followed by 250 mg days 2–5 [25]. None of the primary treatment regimens have been evaluated in randomized controlled trials. In complicated urogenital infections, such as PID and epididymo-orchitis, 400 mg of moxifloxacin should be taken orally for 14 days.

### Resistance

Acquired resistance to first- and second-line treatments (macrolides and fluoroquinolones) is increasing globally, raising concerns due to limited alternative treatments [33]. Macrolide resistance rates are estimated at 30–100% worldwide due to single base mutations in the 23S rRNA gene [34,35]. Fluoroquinolone resistance is increasing, particularly in the Asia-Pacific region, caused by point mutations within the quinolone-resistance-determining regions of the *parC* gene (particularly in positions S83 and D87) [33,36] and with an additional increase in MIC in strains with concurrent mutations in the *gyrA* gene, leading to mutations in M95 and D99 [37]. Fluoroquinolone resistance rates are lower in Europe, but dual resistance to macrolides and fluoroquinolones is not uncommon [33]. No clinical resistance to tetracyclines has been reported, although mutations within the 16S rRNA have been proposed as a potential mechanism [38].

## Pathogenic strategies

### Host range

Humans are the only known host, but non-human primates can be infected and develop symptoms [39].

### Vectors and sylvatic cycle

No vectors or sylvatic cycle has been documented.

### Virulence factors

*M. genitalium* has many virulence mechanisms mediating chronic infection and tissue damage [22]. Adhesion to mucosal surfaces of the reproductive tract is considered the primary virulence factor of *M. genitalium* and a critical step in establishing infection [22]. Adhesion is mediated through the tip structure where the main adhesin MgPa/MG191 (encoded by *mgpB*/*mg191*) is clustered together with an assembly of other proteins [40]. Interestingly, the major immunogens in human infection, MgPa and the accessory adhesin MG192, contain five hypervariable regions scattered throughout the sequence [41]. Sequences with partial homology to these hypervariable regions are found in nine genomic MgPa-related repeats accounting for as much as 4.7% of the total genomic content of this small organism [19]. These repeats serve as a source of genetic variation, helping the organism to escape host immune defence and adapt to various surfaces [42].

Adhesion is also believed to be involved in the internalization of *M. genitalium* [43], a capability that may protect the bacterium from the immune system and contribute to persistence [14].

Currently, *M. genitalium* is not known to produce any exotoxins [22], but it does produce hydrogen peroxide, which may cause some of the ciliopathic effects seen in organ explants [44].

## Epidemiology

### Transmission

The most common mode of transmission is through sexual intercourse, both vaginal and anal. Infected individuals can pass the bacterium to their sexual partners during sexual contact, even if they do not exhibit any symptoms of infection. This includes both heterosexual and homosexual relationships [45,46].

There is evidence to suggest that *M. genitalium* can be transmitted from an infected mother to her child during birth [47]. The clinical relevance for the neonate is unknown.

### Infection

Similar to other STIs, infection can be symptomatic or asymptomatic. Details on the infectious dose or infectivity rate are not known for *M. genitalium*.

### Epidemiology

*M. genitalium* is an STI with a prevalence close to that of *Chlamydia trachomatis* in most studies from high-income countries [48]. Infection rates tend to be higher in sexually active adolescents and young adults, particularly in MSM groups and those attending sexual health clinics [49]. Infection is most prevalent in men aged 25–34 and women aged 16–19, and overall prevalence in high-income counties is estimated to be 1.3% compared with 3.9% in low- and middle-income counties [50].

### Risk groups

Risk groups include sexually active young adults and adolescents, especially those with multiple sexual partners, individuals with other STIs (specifically HIV) and MSM [50]. *M. genitalium* has been shown to increase the risk of HIV acquisition and transmission [51], due to the sustained inflammation.

### Prevention

As with other STIs, prevention is primarily through safe sex practices, including barrier contraceptives, limiting sexual partners and regular STI screenings [49]. At present, no vaccine is under development.

## Open questions

- What is the natural history of *M. genitalium* infections in terms of spontaneous clearance and development of sequelae in symptomatic and asymptomatic individuals?
- Is *M. genitalium* responsible for preterm birth and other adverse neonatal outcomes? If so, how do we treat pregnant women with macrolide-resistant infections?
- What is the proportion of upper genital tract infection (PID and epididymitis) attributable to *M. genitalium?*
- Why is doxycycline only 30% effective when the vast majority of isolates have a MIC that would be considered susceptible?
- How do we treat the increasing numbers of pan-resistant *M. genitalium*?
- Can we develop an axenic culture medium for reliable recovery of *M. genitalium* from clinical specimens?

## Abbreviations

BASHH: British Association for Sexual Health & HIV
MRMs: macrolide resistance mutations
MSM: men who have sex with men
NAAT: nucleic acid amplification tests
NGU: non-gonococcal urethritis
PID: pelvic inflammatory disease
STIs: sexually transmitted infections
